# Phosphorylation of ‘SDT-like’ motifs in ATRX mediates its interaction with the MRN complex and is important for ALT pathway suppression

**DOI:** 10.1098/rsob.240205

**Published:** 2024-12-11

**Authors:** Tomas Goncalves, Harshangda Bhatnagar, Siobhan Cunniffe, Richard J. Gibbons, Anna M. Rose, David Clynes

**Affiliations:** ^1^ MRC Molecular Haematology Unit, Weatherall Institute of Molecular Medicine, University of Oxford, Oxford OX3 9DS, UK; ^2^ Department of Paediatrics, University of Oxford, Oxford OX3 9DU, UK; ^3^ Department of Oncology, University of Oxford, Oxford OX3 7DQ, UK

**Keywords:** ATRX, MRN, alternative lengthening of telomeres

## Abstract

Approximately 10–15% of human cancers are telomerase-negative and maintain their telomeres through a recombination-based process known as the alternative lengthening of telomeres (ALT) pathway. Loss of the alpha-thalassemia/mental retardation, X-linked (ATRX) chromatin remodeller is a common event in ALT-positive cancers, but is generally insufficient to drive ALT induction in isolation. We previously demonstrated that ATRX binds to the MRN complex, which is also known to be important in the ALT pathway, but the molecular basis of this interaction remained elusive. Here, we demonstrate that the interaction between ATRX and MRN is dependent on the N-terminal forkhead-associated and BRCA1 C-terminal domains of NBS1, analogous to the previously reported NBS1–MDC1 interaction. A number of conserved ‘SDT-like’ motifs (serine and threonine residues with aspartic/glutamic acid residues at proximal positions) in the central unstructured region of ATRX were found to be crucial for the ATRX–MRN interaction. Furthermore, treatment with a casein kinase 2 inhibitor prevented the ability of ATRX to bind MRN, suggesting that phosphorylation of these residues by casein kinase 2 is also important for the interaction. Finally, we show that a functional ATRX–MRN interaction is important for the ability of ATRX to prevent induction of ALT hallmarks in the presence of chemotherapeutically induced DNA–protein crosslinks, and might also have implications for individuals with ATR-X syndrome.

## 1. Introduction

To divide indefinitely, cancers need to maintain their telomeres and 10–15% of cancers do this by activating the alternative lengthening of telomeres (ALT) pathway, a specialized form of break-induced replication (BIR) that occurs in the G2- and M-phases of the cell cycle [1,2]. A significant proportion of ALT-positive cancers have mutations in the *ATRX* gene, which encodes a multifunctional protein with roles in chromatin remodelling, genome stability and replication fork processivity. It is thought that telomeric replication stress, in combination with ATRX loss, is a potent trigger of the ALT pathway, through replication fork collapse [2–5]. Ectopic ATRX expression is sufficient to suppress ALT activity; however, its loss alone is generally insufficient to drive ALT induction [6,7]. We recently demonstrated that one form of replication stress that is a potent inducer of ALT in ATRX-null cells is the formation of DNA–protein crosslinks (DPCs) [8].

ATRX has been proposed to have multiple roles during DNA replication, including promoting fork protection and potentiating fork restart [9–12]. Importantly, DNA fibre analysis has demonstrated that stalled replication forks in heterochromatic regions require ATRX for protection, with ATRX depletion leading to excessive fork degradation [11].

It has previously been demonstrated by multiple methods that ATRX interacts with the MRN complex, a protein complex that has a multitude of functions in genome stability and replication [9,10]. It has also been shown that ATRX co-localizes with MRN foci and, of note, this nuclear co-localization occurred exclusively in cells that stained positive for proliferating cell nuclear antigen (PCNA), suggesting the ATRX–MRN interaction occurs during replication [10]. The MRN complex is made up of two MRE11 subunits and two RAD50 subunits which form a core symmetrical hetero-tetrameric MR complex that is stabilized by NBS1 protein subunits [13–15]. Interestingly, chemical inhibition of the MRN complex reversed the fork defect seen in ATRX knockdown cells, suggesting that ATRX might prevent MRE11-dependent degradation at stalled replication forks [11].

Crucially, when MRN is depleted, the ALT pathway is inhibited—this suggests that the ATRX–MRN interaction might be important for regulating ALT [16,17]. It has also been shown that the assembly of functional ALT-associated promyelocytic leukaemia (PML) nuclear bodies (APBs) requires the MRN component NBS1 [18]. Furthermore, ectopic expression of ATRX in U2OS cells results in loss of the MRN complex at telomeres and APBs [6]. We thus wanted to understand the molecular basis of the ATRX–MRN interaction.

By carrying out co-immunoprecipitation pulldowns, we showed that the forkhead-associated (FHA) and BRCA1 C-terminal (BRCT) domains within NBS1 as well as ‘SDT-like’ residues within ATRX are vital in mediating this interaction, permitting us to generate a separation-of-function mutant of ATRX. Using this mutant, we show that the ATRX–MRN interaction is important in suppressing ALT activity in the presence of DPCs. The results presented here will be vital for future studies to determine the exact mechanisms by which the ATRX–MRN interaction regulates ATRX biology.

## 2. Results and discussion

**Figure 1.**
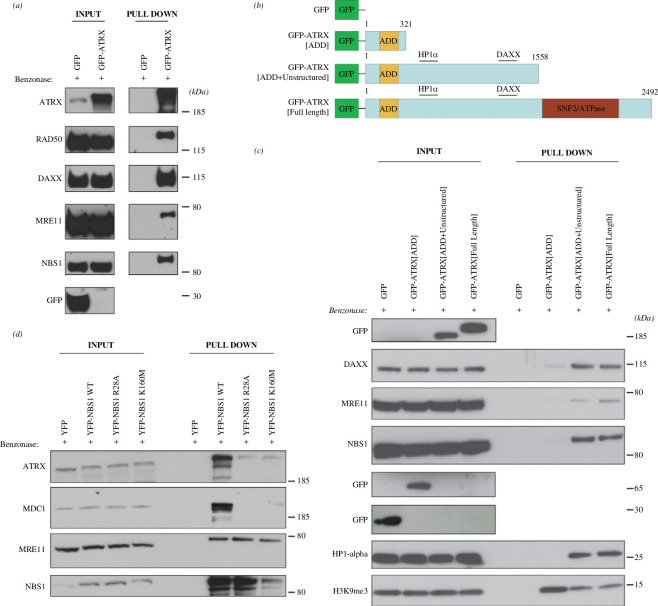
ATRX interacts with the FHA and BRCT domains of NBS1 through its central unstructured region. (*a*) GFP-pulldowns from HEK-293FT cells transfected with constructs containing GFP or the GFP-tagged ATRX construct. The pulldown was repeated three times with similar results. (*b*) Schematic of the GFP-tagged ATRX constructs used in the GFP–ATRX truncation pulldowns, generated by introducing premature stop codons at the indicated residues through site-directed mutagenesis. (*c*) GFP-pulldowns from HEK-293FT cells transfected with the constructs in (*b*). The pulldown was repeated three times with similar results. (*d*) YFP-pulldowns from HEK-293FT cells transfected with constructs containing YFP or the YFP-tagged wild-type, R28A or K160M NBS1 constructs.

We carried out co-immunoprecipitation pulldowns by overexpressing a GFP-tagged ATRX construct in HEK-293FT cells and confirmed that, using this approach, we could detect an association between ATRX and all three members of the MRN complex, that is MRE11, RAD50 and NBS1, as well as the well-characterized ATRX binding partner DAXX (figure 1*a*). ATRX is a large protein that contains an N-terminal ADD (ATRX-DNMT3-DNMT3L) domain, which allows it to bind heterochromatin and a C-terminal SNF2/ATPase domain, which gives it its ATP binding and hydrolysis activity [19]. To narrow down the potential MRN binding sites within ATRX, we generated a series of GFP-tagged ATRX truncation constructs by introducing new stop codons upstream of the natural ATRX stop codon through site-directed mutagenesis (SDM)—one downstream of the ADD domain and another upstream of the SNF2/ATPase domain (figure 1*b*). This generated two truncated ATRX proteins, one very short protein consisting of just the ADD domain (ATRX[ADD]) and a longer protein which comprised both the ADD and unstructured domains (ATRX[ADD + unstructured]) (figure 1*b*). As expected, neither HP1α nor DAXX could strongly interact with the ATRX[ADD] protein product, consistent with the fact that their binding domains have previously been mapped to amino acid residues downstream of this truncated form [20,21]. Meanwhile, ATRX[ADD] could bind the H3K9me3 mark on the histone H3 tail, as previously demonstrated [22,23] (figure 1*c*). Importantly, we found that the MRN complex interacted with ATRX through its central unstructured region, as both MRE11 and NBS1 were found to associate with the ATRX[ADD+unstructured] protein product, but not the ATRX[ADD] product (figure 1*c*).

Within the MRN complex, NBS1 acts as a phosphoprotein-binding and adapter subunit via its N-terminal FHA and BRCT domains [24]. To test whether ATRX was binding to NBS1 through these domains, reverse pulldowns were carried out with wild-type yellow fluorescent protein (YFP)-tagged NBS1, along with R28A and K160M mutant forms that remove crucial phospho-binding residues in the FHA and BRCT1 domains, respectively [24]. Both wild-type and mutant forms of NBS1 could pulldown MRE11; however, like MDC1, ATRX failed to associate with the R28A and K160M mutant constructs, strongly suggesting that ATRX binds to the MRN complex through both the FHA and BRCT domains of NBS1 (figure 1*d*).

**Figure 2.**
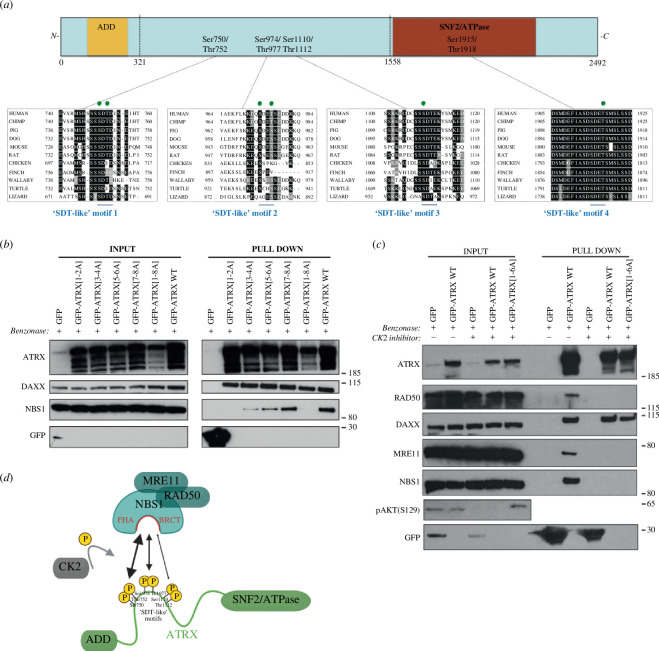
Conserved ‘SDT-like’ motifs phosphorylated by CK2 mediate the ATRX–MRN interaction. (*a*) Schematic highlighting the location of the ‘SDT-like’ motifs within ATRX; multiple sequence alignments of the ‘SDT-like’ motifs are shown below. Identical residues are shaded in black while conservative residues are shaded in grey. Residues that have evidence of phosphorylation according to PhosphoSitePlus have a ‘P’ in a green circle above them. (*b*) Representative GFP-pulldowns from HEK-293FT cells transfected with the indicated GFP constructs. The pulldown was repeated three times with similar results. (*c*) Representative GFP-pulldowns from HEK-293FT cells transfected with the indicated GFP constructs in the presence or absence of the CK2 inhibitor CX-4945. The pulldown was repeated twice with similar results. pAKT(S129) is used as a positive control for the CK2 inhibitor. (*d*) Schematic showing the molecular basis of the ATRX–MRN interaction. Created with BioRender.com.

Proteins that bind to NBS1 through the FHA and BRCT domains, such as MDC1, Treacle and PHRF1, have been shown to do so through ‘SDT-like’ motifs; these having serine and threonine residues with aspartic/glutamic acid residues at proximal positions [25–27]. The ATRX protein sequence was analysed and a series of well-conserved ‘SDT-like’ motifs were identified (figure 2*a*). To test whether these residues were important for the ATRX–MRN interaction, the serine and threonine residues in each of the four candidate ‘SDT-like’ motifs were mutated to alanine via SDM within the GFP-ATRX plasmid, either individually or in combination (table 1).

**Table 1.** Location of ‘SDT-like’ motifs within the ATRX protein, with corresponding SDM-generated constructs used in assays.

‘SDT-like’ motif	region of ATRX	mutant construct
‘SDT-like’ 1	unstructured	GFP-ATRX[1–2A]
‘SDT-like’ 2	unstructured	GFP-ATRX[3–4A]
‘SDT-like’ 3	unstructured	GFP-ATRX[5–6A]
‘SDT-like’ 4	SNF2-ATPase	GFP-ATRX[7–8A]
‘SDT-like’ 1–4	—	GFP-ATRX[1–8A]

When all four SDT-like motifs were mutated in combination, the interaction between ATRX and any of the three members of the MRN complex was completely abolished (figure 2*b*). Importantly, the interaction with DAXX was unaffected, suggesting that overall protein stability is maintained when mutating these motifs. When looking at the motifs individually, it was shown that the C-terminal ‘SDT-like’ motif, located in the highly structured SNF2/ATPase domain, was not important in mediating the ATRX–MRN interaction, while mutating any of the other three ‘SDT-like’ motifs reduced the strength of the ATRX–MRN interaction to varying degrees, with the first motif appearing to be the most important (figure 2*b*). This suggests that Ser750 and Thr752 (‘SDT-like’ 1) are the most crucial residues in mediating the ATRX–MRN interaction. Previous work has shown that these ‘SDT-like’ motifs are reminiscent of the preferred consensus sequence for casein kinase 2 (CK2) [28]. Carrying out the pulldown in the presence of CX-4945, a CK2 inhibitor, completely abrogated the ATRX–MRN interaction, confirming that CK2 is vital in mediating this interaction (figure 2*d*). Together, these results provide a detailed overview on the molecular basis of the ATRX–MRN interaction (figure 2*e*).

**Figure 3.**
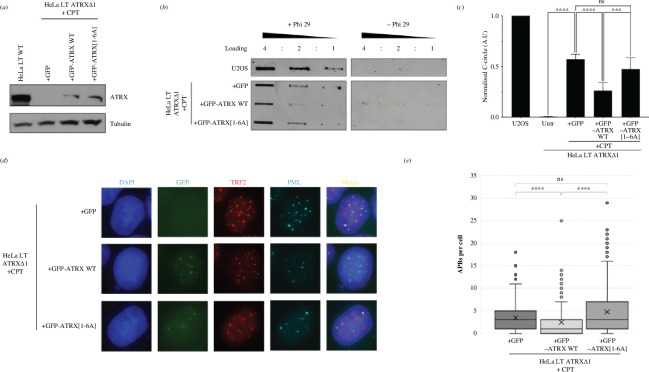
The ATRX–MRN interaction is important for ATRX to suppress ALT pathway induction following DPC formation. (*a*) Immunoblot showing expression levels of ATRX following transient transfection of GFP-ATRX WT and GFP-ATRX[1–6A] constructs in HeLa LT ATRXΔ1 cells. For comparison, HeLa LT wild-type cells were also included. (*b*) Representative C-circle assay blot of HeLa LT ATRXΔ1 transiently transfected with GFP, GFP-ATRX WT or GFP-ATRX[1–6A] constructs and then treated with CPT. (*c*) Quantification of B, one-way ANOVA, *n* = 6. (*d*) Representative immunofluorescence images of HeLa LT ATRXΔ1 cells treated with CPT and transiently transfected with GFP, GFP-ATRX WT or GFP-ATRX[1–6A] constructs, stained for TRF2 and PML (*e*) Quantification of D > 300 cells analysed across three biological replicates, Kruskal–Wallis test.

The role of the ATRX–MRN interaction in suppressing the ALT pathway was next investigated. We recently demonstrated that in ATRX-null HeLa LT cells, exposure to DPC-forming chemotherapeutics, such as the TOP1 poison camptothecin (CPT), resulted in induction of ALT activity [8]. We transfected the HeLa LT ATRXΔ1 cells with either GFP-ATRX[WT] or GFP-ATRX[1–6A] constructs through transient transfection and then exposed the cells to 50 nM CPT for 48 h (figure 3*a*). As expected, GFP-ATRX[WT] could significantly reduce the induction of two canonical ALT hallmarks: C-circles and APBs (figure 3*b*–*d*). However, GFP-ATRX[1–6A] failed to reduce the levels of ALT hallmarks, strongly suggesting the ATRX–MRN interaction is crucial in the ability of ATRX to suppress ALT in the presence of DPCs, such as TOP1cc (figure 3*b*–*d*).

Finally, we considered the role of the identified ATRX–MRN interaction in ATR-X syndrome, the congenital multi-system syndrome associated with germline loss of ATRX gene function. Analysis of ClinVar data identified two reported germline changes in three affected individuals (SCV000762428, SCV001764152 and SCV003455660), which affected the serine 750 residue within ‘SDT-like’ motif 1, which we have determined to be the most important mediator of the ATRX–MRN interaction. This variant has previously been classified as a variant of unknown significance, due to the fact that the residue falls in an unstructured region, meaning there was insufficient evidence to determine the role of the variant in disease; however, our data presented herein would strongly suggest that this change is pathogenic through interruption of the ATRX–MRN interaction.

In this work, the molecular basis of the ATRX–MRN interaction was elucidated for the first time. It was shown that ATRX could interact with the MRN complex independently of MDC1 and, within the MRN complex, ATRX bound directly to NBS1. This supports the results of a previous proteomic screen which specifically identified the NBS1 component of the MRN complex as an interaction partner [29]. The FHA/BRCT domains within NBS1 were both crucial in mediating the interaction, while it was discovered that ‘SDT-like’ residues within the central unstructured regions of ATRX, were vital for the interaction, with the Ser750/Thr752 residues (‘SDT-like’ 1) appearing to be most important. As inhibition of CK2 also prevented the interaction, it is likely that the ATRX–MRN interaction is dependent on phosphorylation of the ‘SDT-like’ motifs by CK2. However, it is important to note that CK2 has many physiological substrates and targets a multitude of proteins and pathways which may indirectly inhibit the ATRX–MRN interaction. Future work to confirm the requirement of CK2 in phosphorylating the SDT-like motifs within ATRX should be carried out in the future, for example, by introducing phospho-mutants into ATRX or using recombinant protein approaches.

This work also presents insights into the role of the ATRX–MRN interaction in suppression of the ALT-pathway. Through transient transfections of GFP-tagged products, either wild-type ATRX, or mutant ATRX (mutant SDT-like motifs in the unstructured region; ATRX[1–6A]) was re-expressed in ATRX-null HeLa LT cells. Treatment of ATRX-null HeLa LT cells with DPC-forming chemotherapeutic agents, such as camptothecin, is a potent trigger for ALT pathway induction. Re-expression of wild-type ATRX could significantly reduce the induction of ALT hallmarks following treatment of the cells with DPC-forming chemotherapeutic agents; however, the ATRX[1–6A] mutant failed to do so. This strongly suggests that the ATRX–MRN interaction plays a fundamental role in the ability of ATRX to suppress ALT activation in cells following replication stress, such as DPC formation.

Although the ATRX–MRN interaction has been well characterized here, some important question still remain. First of all, it is unclear how the ATRX–MRN interaction is important in suppressing ALT. A previous report using chromatin immunoprecipitation analysis and immunofluorescence experiments indicated that ectopic expression of ATRX in U2OS cells leads to loss of the MRN complex from telomeres and APBs [6] This raises the intriguing possibility that ATRX might, therefore, be sequestering MRN away from APBs, preventing excessive nucleolytic degradation of stalled forks. However, as the MRN complex has been shown to be essential for ALT activity, this could also be a secondary effect following ALT pathway inhibition by ATRX expression.

Alternatively, it has previously been speculated that ATRX, MRN and Fanconi anaemia complementation group D2 (FANCD2) might be recruited to stalled replication forks, where they may form a ‘fork restart super-complex’ which recruits CtIP and promotes fork restart [12]. In support of this theory, this super-complex would have a molecular weight of approximately 1.1 MDa, which is equivalent to a previously discovered, but until now uncharacterized FANCD2-containing protein complex [30]. Therefore, rather than sequestering MRN from the telomere, ATRX could instead be playing a role in regulating MRE11 activity along with FANCD2 to promote effective fork restart.

Finally, the ATRX–MRN interaction is one of very few stable ATRX protein–protein interactions, along with the ATRX–DAXX interaction, which have been identified [6,9,29]. Given that ATRX plays multiple roles in the cell, it is possible that such a strong interaction might be important in some of these other functions, beyond ALT suppression. Mutations affecting serine 750 were identified in three individuals with ATR-X syndrome, suggesting that the ATRX–MRN might well have roles in the mediation of the protein’s other functions. However, further testing is required, including assessment of perturbation of ATRX’s other roles upon loss of the various unstructured region ‘SDT-like’ motifs identified herein.

In conclusion, this work presents the molecular basis for the interaction between the MRN-complex and ATRX. The interaction—mediated directly through the NBS1 component of the MRN—is dependent on phosphorylation of SDT-like motifs in the central, unstructured ATRX region. This interaction was shown to be important for the suppression of ALT pathway activity, which has important implications for our understanding of this critical cancer pathway. Furthermore, it might explain the presentation of ATR-X syndrome in individuals harbouring germline mutations within key serine–threonine residues.

## 3. Material and methods

### 3.1. Cell culture conditions

The U2OS and HEK-293FT cell lines were acquired from American Type Culture Collection (ATCC), while the HeLa LT cell line was a gift from Roderick O’Sullivan (University of Pittsburgh, USA). The generation of the CRISPR/Cas9-mediated ATRX knockouts were described previously [8]. Cells were grown in a 5% CO_2_ 37°C incubator with Dulbecco’s modified Eagle’s medium (DMEM) media supplemented with 10% fetal bovine serum, 1% l-glutamine and 1% PenStrep (all Gibco).

### 3.2. Plasmid construction and cloning

The GFP-ATRX WT plasmid was acquired from Addgene (plasmid no. 45444), described in [31], while the GFP empty vector and the YFP-NBS1[WT], YFP-NBS1[R28A] and YFP-NBS1[K160M] were gifts from Andrew Blackford (University of Oxford, UK). The GFP-ATRX[ADD] and GFP-ATRX[ADD + unstructured] constructs were generated by inserting premature stop codons, while the GFP-ATRX[1–2A], GFP-ATRX[3–4A], GFP-ATRX[5–6A], GFP-ATRX[7–8A], GFP-ATRX[1–6A] and GFP-ATRX[1–8A] constructs were generating by substituting the Ser/Thr residues to Ala. In both cases, this was achieved through site-directed mutagenesis using the Q5 site-directed mutagenesis kit (New England Biolabs) according to manufacturer’s instructions. Primers used are listed in table 2. Full plasmid sequences are available upon request.

**Table 2.** Primers used for site-directed mutagenesis experiments.

primer	sequence
ATRX[ADD+Unstructured] Fw	5′-TATGGTTATCTAATTGAAACCCC−3′
ATRX[ADD+Unstructured] Rv	5′-TTTCTATGAACCTGCACTAAAG−3′
ATRX[ADD] Fw	5′-GAAGACTAGTTGAAATTGTAATGG−3′
ATRX[ADD] Rv	5′-TTTGGAGAAAATCTGGATG−3′
ATRX[1–2A] Fw	5′-GCTGATATTAATGAAATTCATACAAACCATAAG-3′
ATRX[1–2A] Rv	5′-ATCTGCAGAAGAACTGTGACTCATTC-3′
ATRX[3–4A] Fw	5′-GAAGCTTCTGAAGATGATAAAAAGCAGAGC-3′
ATRX[3–4A] Rv	5′-ATCGGCCTGGTCTTTCTTTAGGAATTTCTCTG-3′
ATRX[5–6A] Fw	5′-GCTGAGAAATATTCCATGAAAGAAGATGG−3′
ATRX[5–6A] Rv	5′-ATCAGCTGATGAACAATCTTGTCTCTTCC-3′
ATRX[7–8A] Fw	5′-GAAGCCTCCATGAGTTTAAGCTCCGATG-3′
ATRX[7–8A] Rv	5′-ATCAGCATCTGAGGCTATAAATTCATCCATAC-3′

### 3.3. GFP/YFP pulldowns

HEK-293FT cells were seeded into 15 cm dishes and transfected with 18 μg of plasmid DNA with Lipofectamine 2000 (Thermo Fisher) according to manufacturer’s instructions. After 18 h, lysates were extracted using IP lysis buffer (50 mM Tris-HCl pH 7.4; 100 mM NaCl; 1 mM MgCl_2_; 10% glycerol; 5 mM NaF; 0.2% IgePal CA-630, 1 × complete protease inhibitor cocktail) and the lysates were treated with 25 U ml^−1^ of Benzonase (Sigma-Aldrich) for 45 min at 4°C. The buffer was then adjusted to a final concentration of 2 and 200 mM NaCl. The lysates were clarified through centrifugation and 2% was taken as the input. The remainder of the lysate was incubated with GFP-trap magnetic agarose beads (ChromoTek) for 2 h at 4°C. The beads were washed four times with IP lysis buffer and then eluted in 2 × SDS protein loading buffer by heating to 95°C for 5 min. Immunoblotting was then performed on the samples.

### 3.4. Immunoblotting

Lysates were prepared as described above. Samples were loaded into precast 4–12% Bis-Tris gels with 1 × 3-(N-morpholino)propanesulfonic acid (MOPS) running buffer (both Thermo Fisher Scientific). Membranes were transferred at a constant current of 30 mA overnight at 4°C onto methanol-activated polyvinylidene fluoride (PVDF) membranes (Millipore) with NuPage transfer buffer (Thermo Fisher Scientific), supplemented with 10% methanol. The next day, the membranes were blocked in 5% milk in PBST (0.1% tween in 1 × phosphate-buffered saline) and then incubated for 1 h at room temperature with the following primary antibodies in 2.5% milk in PBST: rabbit anti-ATRX (Abcam, ab97508, 1 : 1000), mouse anti-RAD50 (Santa Cruz, sc-74460, 1 : 200), rabbit anti-DAXX (Sigma, D7810, 1 : 2000), rabbit anti-MRE11 (Novus Biologicals, NB100-142, 1 : 5000), mouse anti-NBS1 (Santa Cruz, sc-515069, 1 : 200), mouse anti-GFP (Sigma, 11814460001, 1 : 5000), mouse anti-HP1α (Abcam, ab234085, 1 : 500), rabbit anti-H3K9me3 (Abcam, ab9050, 1 : 1000), rabbit anti-MDC1 (Abcam, ab11171, 1 : 5000), rabbit anti-pAKT[S129] (Abcam, ab133458, 1 : 1000), mouse anti-ATRX (Santa Cruz, sc-55584, 1 : 500) and mouse anti-alpha tubulin (Abcam, ab7291, 1 : 50 000). Membranes were then washed three times at room temperature for 10 min with PBST and then incubated with the following horseradish peroxidase (HRP)-conjugated secondary antibodies diluted in 2.5% milk in PBST for 1 h at room temperature: rabbit anti-mouse IgG HRP (Sigma, A9044, 1 : 5000) or goat anti-rabbit IgG HRP (Thermo Fisher Scientific, 31460, 1 : 5000). Membranes were again washed three times for 10 min with PBST and then developed using SignalFire ECL reagent (Cell Signalling) onto X-ray films (Amersham).

### 3.5. Multiple sequence alignments

The ATRX orthologue sequences were acquired from UniProt. Sequences were aligned using T-Coffee [32]. Conserved residues were shaded in black while similar residues were shaded in grey using BoxShade, which can be accessed at https://embnet.vital-it.ch/software/BOX_form.html.

### 3.6. Treatment of cells with drugs

For pulldowns in the presence of the CK2 inhibitor, cells were transfected as described above and 5 h later, the media was replaced with media containing 10 μM of CX-4945. The cells were then incubated with the drug for 18 h before lysates were extracted for pulldowns. For CPT treatment, cells were transiently transfected as described below and then 5 h later, the media was replaced with media containing 50 nM CPT and the cells were incubated for 48 h before harvesting/fixing.

### 3.7. Transient transfection of GFP-ATRX[WT] and GFP-ATRX[1-6A] constructs

HeLa LT ATRXΔ1 cells were seeded out into six-well dishes for DNA extractions or 13 mm #00 thickness glass coverslips in 24-well dishes for indirect immunofluorescence so that they would be approximately 75% confluent at the time of transfection. The following day, cells were transfected with the plasmids using JetPrime transfection reagent (PolyPlus) according to manufacturer’s instructions. Five hours later, the media was replaced with either standard media or media containing CPT as described above and incubated for a further 48 h.

### 3.8. C-circle assay

Genomic DNA was extracted from cells using the PureLink genomic DNA extraction kit (Thermo Fisher Scientific) and quantitated by NanoDrop; 30 ng of DNA was amplified using a thermocycler for 8 h at 30°C followed by 20 min at 65°C in a reaction containing 7.5 U of φ29 polymerase (New England Biolabs), 0.1% Tween-20, 200 µg ml^−1^ recombinant albumin (New England Biolabs) and 1 mM each of dTTP, dGTP and dATP (all New England Biolabs) diluted in nuclease-free water. Negative controls (without φ29 polymerase) and positive controls (U2OS gDNA) were included in each blot. The samples were then diluted with 2 × saline-sodium citrate (SSC) buffer and transferred onto Zeta-Probe membranes (Bio-Rad) using a slot blot filtration manifold in a twofold dilution series. The DNA was then UV crosslinked onto the membranes which were then pre-hybridized with DIG Easy Hyb (Roche) for 20 min at room temperature before being incubated at 37°C for 2 h with 40 nM of a 3′-DIG-labelled [CCCTAA]_5_ probe diluted in DIG Easy Hyb. Membranes were then rinsed twice in MS wash buffer (0.1 M maleic acid, 3 M NaCl, 0.3% Tween-20, pH 7.5) and blocked for 30 min at room temperature in MS blocking buffer (1% milk and 1% BSA in 0.1 M maleic acid, 3 M NaCl, pH 7.5). Anti-DIG-AP Fab fragments (Roche) were then added to the MS blocking buffer (1 : 20 000) and the membranes were incubated for 1 h at room temperature. The membranes were then washed three times for 15 min at room temperature with MS wash buffer and developed using CDP-Star chemiluminescent substrate solution (Roche) for X-ray film detection. Films were scanned and quantified using ImageJ software. Normalized CC levels were expressed as arbitrary units relative to U2OS.

### 3.9. Indirect immunofluorescence for APB analysis

Cells grown on glass coverslips were pre-permeabilized in 0.5% triton X-100 (Sigma) in PBS for 1 min on ice and then fixed with 4% paraformaldehyde (Thermo Fisher Scientific) in PBS for 20 min at room temperature. The cells were washed in PBS at room temperature and then post-permeabilised with 0.5% triton X-100 on ice for 6 min. Cells were washed three times in PBS at room temperature and then blocked for 1 h in 1% BSA in PBS. Blocked cells were then incubated for 1 h at room temperature with the following primary antibodies diluted in 1% BSA in PBS: mouse anti-PML (Santa Cruz, sc-966, 1 : 300), rabbit anti-TRF2 (Novus Biologicals, NB110-57130, 1 : 500). Cells were washed four times with PBST and then incubated for 1 h at room temperature with the following fluorescently labelled secondary antibodies diluted in 1% BSA in PBS: goat anti-rabbit Alexa Fluor 568 (Life Technologies, A11036, 1 : 3000), goat anti-mouse Alexa Fluor 647 (Life Technologies, A32728, 1 : 3000). After three washes in PBST, coverslips were then mounted onto microscope slides with VectaShield containing DAPI and imaged using a DeltaVision widefield microscope at 60× magnification. Images were z-projected using Fiji ImageJ software and image analysis was performed using CellProfiler [33].

### 3.10. Statistical analysis

For analysis of the C-circle data (parametric data), a one-way ANOVA with Welch correction was performed. For analysis of APB data (non-parametric data), an unpaired Kruskal–Wallis test was performed. Sample sizes and replicate numbers are indicated in the figure legends. In each case, significance was considered as *p < 0.05, **p < 0.01, ***p < 0.001, ****p < 0.0001. ns denotes no significance. Statistical analysis was carried out using GraphPad Prism 9 (GraphPad Software Inc.).

## Data Availability

All data generated and/or analysed for this study are included within the published article.
